# The Effect of Adverse Surgical Margins on the Risk of Biochemical Recurrence after Robotic-Assisted Radical Prostatectomy

**DOI:** 10.3390/biomedicines10081911

**Published:** 2022-08-07

**Authors:** Enric Carbonell, Roger Matheu, Maria Muní, Joan Sureda, Mónica García-Sorroche, María José Ribal, Antonio Alcaraz, Antoni Vilaseca

**Affiliations:** 1 Department of Urology, Hospital Clínic de Barcelona, Villarroel 170, 08036 Barcelona, Spain; 2 Clinical Institute of Nephrology and Urology (ICNU), Hospital Clínic de Barcelona, Villarroel 170, 08036 Barcelona, Spain; 3 Department of Surgery and Surgical Specialties, Universitat de Barcelona, Casanova 143, 08036 Barcelona, Spain

**Keywords:** prostate cancer, robotic-assisted radical prostatectomy, biochemical recurrence, positive surgical margins

## Abstract

Positive surgical margins (PSM) after radical prostatectomy are associated with a greater risk of biochemical recurrence (BCR). However, not all PSM harbour the same prognosis for recurrence. We aim to determine the impact of different PSM characteristics and their coexistence on the risk of BCR. This retrospective study included 333 patients that underwent robotic-assisted radical prostatectomy for prostate cancer between 2015–2020 at a single institution. The effect of PSM and their adverse characteristics on the risk of BCR was assessed using Cox proportional hazard models. Kaplan–Meier was used to represent BCR-free survival stratified by margin status. With a median follow-up of 34.5 months, patients with PSM had a higher incidence of BCR, higher risk of relapse and lower BCR-free survival than negative margins (*p* < 0.001). We established as adverse characteristics: PSM length ≥ 3 mm, multifocality and Gleason at margin > 3. PSM ≥ 3 mm or multifocal PSM were associated with an increased risk for BCR compared to favourable margins (HR 3.50; 95% CI 2.05–5.95, *p* < 0.001 and HR 2.18; 95% CI 1.09–4.37, *p* = 0.028, respectively). The coexistence of these two adverse features in the PSM also conferred a higher risk for biochemical relapse and lower BCR-free survival. Adverse Gleason in the margin did not confer a higher risk for BCR than non-adverse margins in our models. We concluded that PSM are an independent predictor for BCR and that the presence of adverse characteristics, such as length and focality, and their coexistence in the PSM are associated with a greater risk of recurrence. Nevertheless, subclassifying PSM with adverse features did not enhance the model’s predictive performance in our cohort.

## 1. Introduction

Positive surgical margins (PSM), which are defined as the presence of tumour cells at the inked margin after radical prostatectomy (RP), are consistently associated with a greater risk of biochemical recurrence (BCR) [1,2,3,4,5,6]. The published rate of PSM after RP varies between 10–46% [3,7,8,9,10], and their presence depends on various factors, including disease location and stage, patient’s characteristics, surgical technique and surgeon’s experience, among others [6]. The impact of PSM on more robust oncologic endpoints as clinical recurrence or prostate cancer-specific mortality is uncertain [3,11]. Therefore, the ideal management of patients with PSM is controversial. Randomised trials showed the benefit of adjuvant radiation therapy (ART) in patients with PSM and the coexistence of other high-risk features such as seminal vesicle involvement or extra-prostatic extension [12,13,14,15]. However, PSM alone does not seem to be sufficient criteria to initiate ART and may lead to overtreatment and toxicity [11,16]. Thus, in the case of PSM after RP, the European Association of Urology guidelines recommend ART for patients with other high-risk features such as high pathological ISUP or non-organ-confined disease [17]. Currently, ongoing trials are trying to assess the role of close surveillance and early salvage radiotherapy to avoid ART and overtreatment in patients with adverse pathology after RP, including PSM [18,19,20]. The collaborative ARTISTIC meta-analysis showed no benefit of ART compared to early salvage radiotherapy in terms of event-free survival, suggesting early salvage radiotherapy as the standard of care [21].

Nevertheless, not all PSM carry the same risk for BCR and clinical recurrence. In this setting, Martini et al. have defined unfavourable or clinically meaningful PSM that could better predict BCR and stronger oncological outcomes and could justify additional therapies based on PSM characteristics [22]. In previous studies, PSM risk stratification has been assessed, focusing on PSM characteristics such as length, location, number of positive margins and Gleason at the margin. There is limited evidence about how these characteristics impact BCR and only a few studies have defined the effect of some of them [8,23,24,25,26,27,28,29]. In recent studies, PSM subclassification improved the accuracy of predictive models for BCR [30,31]. Moreover, Morizane et al. recently introduced a risk stratification model classifying PSM into good-, intermediate- and poor-risk groups for BCR depending on margin’s length and Gleason [32]. However, the combined prognostic impact on BCR of unfavourable PSM features regarding length, focality and Gleason at the margin has not been previously described.

The primary aims of the present study were to describe PSM characteristics in patients that underwent robotic-assisted radical prostatectomy (RARP) and assess the prognostic value of the different PSM characteristics individually and in combination to establish those patients with the greatest risk of BCR.

## 2. Patients and Methods

### 2.1. Patient Selection

After receiving approval from our institutional review board (IRB) (HCB/2022/0043), data from men that underwent RARP from 2015–2020 at our institution were retrospectively collected and introduced in our database (*n* = 442). Informed consent was waived because of the retrospective nature of the study and the analysis used anonymous clinical data. Men treated with RARP for localised or locally advanced disease and had full information available regarding margins status, follow-up and recurrence were included. We excluded patients who received neoadjuvant or adjuvant treatment (*n* = 32), patients with less than 6 months of follow-up (*n* = 36) or patients followed-up in other institutions (*n* = 41). Finally, 333 men were included in the study.

### 2.2. Surgical Technique

The surgical procedures were performed by eight different urologists at our institution. All the patients underwent the same surgical approach. Robotic-assisted radical prostatectomy was performed through a six-port transperitoneal approach using the four-arm Da Vinci Si (Intuitive Surgical, Sunnyvale, CA, USA) Robotic Surgical System following previously described techniques [33,34]. Modifications of the technique, including neurovascular bundle preservation or lymphadenectomy, were performed depending on the disease stage and location of the tumour.

### 2.3. Pathology Examination

Pathologic specimens were evaluated by expert uropathologists at our institution. The pathology report included: prostate weight and volume, pathologic stage (TNM), pathologic ISUP, tumoral focus including length and location, lymphovascular and perineural invasion, surgical margin status, PSM location/s, PSM length (multiple lengths were reported if multifocality was assessed) and Gleason at the PSM. PSM were defined as the presence of tumour cells at the inked margin. PSM length was defined as the total length of the tumour in contact with the inked margin. If multifocal PSM were detected, the length of all tumoral margins were added to the report and the longest was considered for analysis. The highest Gleason grade at the PSM was stated.

### 2.4. Study Covariates and Outcomes

Data were collected retrospectively in an ethics-approved, secure research database and included baseline clinicopathological parameters (demographics, preoperative PSA, prostate biopsy, clinical stage), surgical data, and prostatectomy specimen information, as previously described. Patient follow-up with PSA and physical examination was performed according to our institution protocol; at months 3, 6 and 12 during the first year after surgery, at the 6-month interval for 2–3 years and annually thereafter. The main outcome of the study was biochemical recurrence-free survival depending on surgical margin status and characteristics. BCR was defined as two consecutive serum PSA values ≥ 0.2 ng/mL.

### 2.5. Statistical Analysis

Demographic and clinicopathological variables were assessed using frequencies and proportions for categorical variables while medians and interquartile ranges (IQR) for continuous variables. These variables were compared, stratified by margin status, using the Chi-square test for categorical variables and Mann–Whitney U test for continuous variables. The impact of margin status (positive vs. negative) and PSM characteristics, individually and combined, on the risk of BCR was assessed using univariable and multivariable Cox proportional hazard regression models. The adjustment was made for covariates known as prognosis factors for disease recurrence, including preoperative PSA, pathologic stage (pT) and pathologic ISUP. The adverse margin characteristics were selected according to evidence from previous studies; PSM length ≥ 3 mm, multifocality and Gleason at the margin > 3 [7,8,25,26,27,35,36]. Biochemical recurrence-free survival was defined as the time between surgery and the date of BCR. Patients alive without BCR at the last follow-up visit were censored in the analysis. The c-indices were assessed to determine the model’s performance and discriminative ability. The bootstrapping method with 1000 bootstrap samples for each model was performed for model validation and to evaluate optimism-corrected change in discrimination. Biochemical recurrence-free survival was represented using the Kaplan–Meier method. Survival curves were represented stratified by margin status and adverse characteristics and compared using the two-sided log-rank test. A double-sided *p* value < 0.05 was considered statistically significant. All data were analysed using SPSS version 25.0 software (SPSS, Chicago, IL, USA).

## 3. Results

### 3.1. Descriptive Characteristics

Baseline clinicopathological variables of the cohort and stratified by margin status are shown in Table 1. The median age at the time of the surgery was 65 years (interquartile range (IQR) 59–68.5 years). Most of the patients presented organ-confined disease (75.1%) with a pathologic ISUP ≤ 3 (89.8%) and a median preoperative PSA of 6 ng/mL (IQR 4.8–8.5 ng/mL). PSM were reported in 124 (37.2%) RARP specimens. PSM were more likely in patients with advanced disease stages in terms of extracapsular extension (33.1% vs. 20.1%, *p* = 0.008). However, there was no difference between groups regarding ISUP grade in the final pathology (*p* = 0.149). We grouped ISUP 2 and 3 for the final analysis considering EAU risk groups for BCR and that results from the separate analysis did not differ. Prostate gland volumes in RARP specimens were smaller in the PSM group compared to men with negative surgical margins (NSM) (42.5 grs vs. 55 grs; *p* < 0.001). The median length of the PSM (Table 2) was 3.5 mm (IQR: 2–6 mm). The commonest PSM locations were the posterior region (31.5%) and the apical region (25.8%). Most of the PSM were unifocal (73.4%) and had a Gleason 3 at the margin (59.7%). PSM in pT3 specimens were mostly not located at the extracapsular extension location. 

With a median follow-up of 34.5 months (IQR 26.3–47.0 months), patients with PSM had higher BCR rates than patients with NSM (32.3% vs. 9.6 %; *p* < 0.001).

### 3.2. Impact of PSM and Their Adverse Characteristics on the Risk of BCR

Pathologic stage, pathologic ISUP and PSM and their adverse characteristics were predictive of BCR on univariable analysis (all *p* < 0.05) (Supplementary Table S1). On Cox regression multivariable analysis (Table 3) adjusted for known predictors of BCR as preoperative PSA, pathological stage and pathologic ISUP, PSM showed to be an independent predictor for BCR (hazard ratio (HR) 3.71; 95% confidence interval (CI) 2.13–6.47, *p* < 0.001). When stratifying PSM by their adverse characteristics, patients with PSM ≥ 3 mm or multifocal PSM were at an increased risk of BCR (HR 4.51; 95% CI 2.49–8.17 and HR 4.16; 95% CI 1.90–9.09 respectively, *p* < 0.001) and harboured higher risk for biochemical relapse compared to favourable surgical margins (HR 3.50; 95% CI 2.05–5.95, *p* < 0.001 and HR 2.18; 95% CI 1.09–4.37, *p* = 0.028 respectively). In our cohort, Gleason at the margin > 3 was not statistically associated with a higher risk for recurrence compared with non-adverse margins (HR 2.00; 95% CI 0.94–4.27, *p* = 0.072). After combining adverse PSM features regarding length and focality, PSM ≥3 mm and multifocal strongly predicted BCR (HR 4.66; 95% CI 2.08–10.42, *p* < 0.001) and conferred a higher risk for BCR in comparison with favourable surgical margins (HR 2.43; 95% CI 1.18–4.99, *p* = 0.016). PSM with all adverse features; ≥ 3 mm, multifocal and with Gleason at margin > 3, emerged as an independent predictor for BCR with the highest risk associated in our models (HR 5.98; 95% CI 1.86–19.24, *p* = 0.003). However, in our cohort, PSM with all adverse features didn’t reach a significantly increased risk of BCR when compared to non-adverse margins (HR 2.99; 95% CI 0.99–9.02, *p* = 0.052), probably due to the low representation of this type of margin in our cohort.

The model’s discrimination considering surgical margin status (positive vs. negative) was 0.738. A non-significant increase in discrimination was obtained when adverse characteristics of the PSM were added to the prediction model for BCR (Table 3).

**Figure 1:**
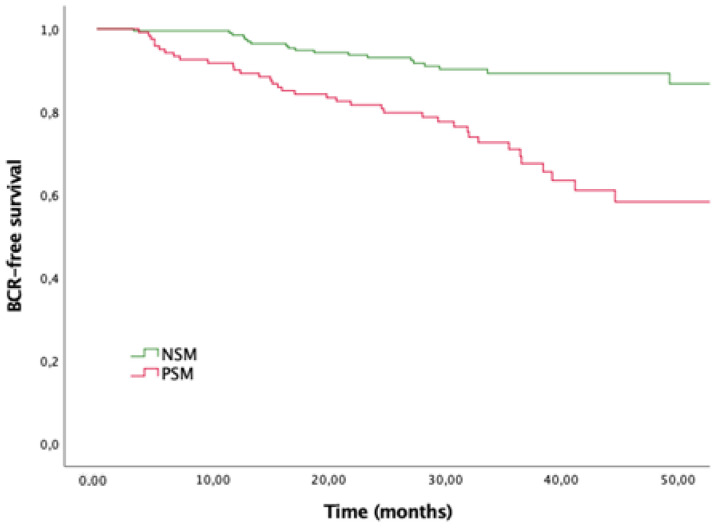
Kaplan-Meier curves for biochemical-recurrence stratified by margin’s status. Log-rank *p* < 0.001.

On Kaplan–Meier survival analysis, men with PSM after RARP (Figure 1) showed lower overall BCR-free survival (BFS) compared with men with NSM (*p* < 0.001), with BFS rates at 36 months of follow-up of 67.5% for PSM vs. 89.3% for NSM. 

**Figure 2:**
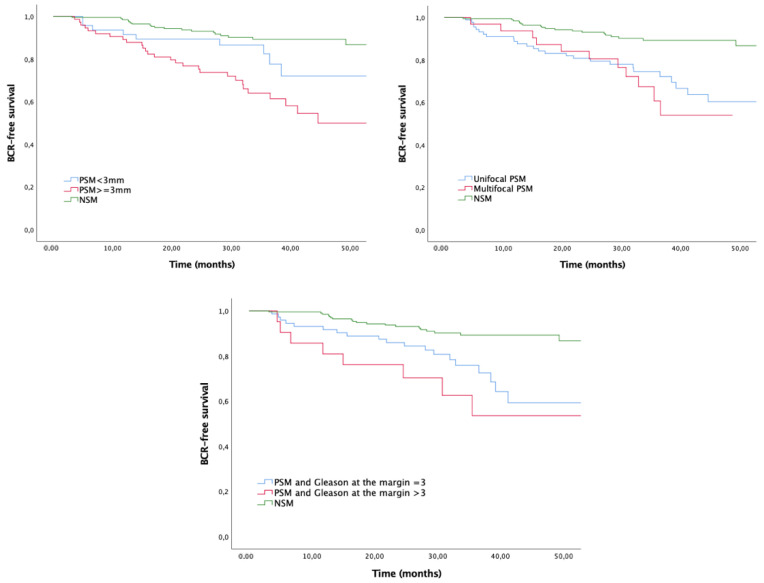
Kaplan–Meier curves for biochemical recurrence stratified by margin’s characteristics. Log-rank *p* < 0.001.

**Figure 3:**
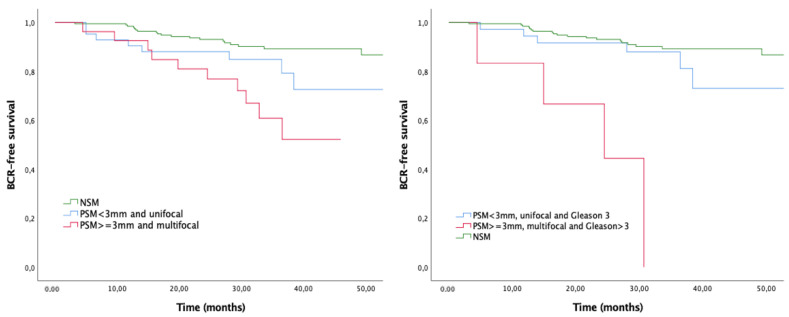
Kaplan–Meier curves for biochemical recurrence stratified by combined adverse margin characteristics. Log-rank *p* < 0.001.

When survival curves were stratified by adverse PSM characteristics, all of them conferred lower BFS compared with NSM (all *p* < 0.001) (Figure 2). BFS rates at 36 months were 64% for PSM ≥ 3 mm, 60.7% for multifocal PSM and 53.6% for PSM with Gleason > 3. The coexistence of adverse features in the PSM showed decreased BFS as well (*p* < 0.001) (Figure 3). 

Regarding PSM location (Supplementary Table S2), apical and posterior PSM were independent predictors of recurrence (*p* ≤ 0.001). 

Finally, we performed a subgroup analysis to assess the impact of PSM and their features in patients with favourable pathology after RARP (pT2 and ISUP ≤ 3) (Table 4). In these patients, PSM and their adverse characteristics, alone and combined (length and focality), predicted well BCR (all *p* ≤ 0.001). There were no patients in this subgroup with PSM featuring all three adverse characteristics (≥3 mm, multifocal and with Gleason at margin > 3).

## 4. Discussion

The presence of PSM after RP is associated with a greater risk of BCR [1,2,3,4,5,6]. However, most of the studies assessing the impact of PSM on recurrence did not consider margins’ characteristics nor their impact on stronger oncologic outcomes. Data regarding the effect of PSM on clinical recurrence or prostate cancer-specific mortality did not show PSM as an independent predictor of robust clinical endpoints [1,11,16,37]. Nevertheless, we cannot consider all PSM as equal, taking into account that not all of them own the same risk of recurrence. In this context, some studies have tried to define unfavourable or adverse PSM characteristics focusing on PSM length, location, focality or Gleason at the margin and their impact on BCR [8,23,24,25,26,27,28,29,30]. In this setting, Martini et al. reported higher clinical recurrence with higher metastases rates in patients with unfavourable PSM (multiple positive margins or ≥3 mm) compared to patients with favourable PSM, supporting the hypothesis that PSM features matter [22]. Furthermore, recently published studies assessed if subclassifying PSM could add value to predictive models for BCR and concluded that this could improve clinical decision-making for patients with PSM [30,31]. The present study aimed to describe the impact of different PSM characteristics on the risk of BCR in a series of RARP performed at our institution. Additionally, we assessed the combined effect of adverse PSM characteristics, including length, focality and Gleason at the margin, on the risk of BCR and established new insights to address the classification or risk stratification of PSM for BCR.

We reported a PSM rate of 37.2%. The rate of PSM in the literature varies enormously and depends on several factors such as disease stage, pathologic assessment, surgical technique or surgical experience, and it ranges between 10–46% [10,38,39]. We reported relatively high PSM rates in our series due to several reasons; we included patients with organ and non-organ confined disease and, mainly, due to the surgical learning curve as we started our robotic prostatectomies program in 2015 and surgeries were performed by different urologists. PSM rate decreased over the inclusion period (data not shown). 

In our cohort, patients with PSM had higher incidence of BCR (32.3% vs. 9.6%, *p* < 0.001), lower BFS (*p* < 0.001) and had a 3.71-fold increased risk of BCR. These findings support previous evidence [1,2,3,4,5,6]. To stratify PSM into different risk categories, studies have tried to assess the effect of different PSM features on BCR, mainly length, focality and Gleason at the margin. Length of the PSM and its impact on BCR has been mainly assessed as a categorical variable in previous studies (<1 mm vs. ≥1 mm and <3 mm vs. ≥3 mm) and BCR rates showed to increase with larger PSM [23,24,40]. We defined a cut-off length of ≥ 3 mm, which is related to poorer outcomes [7,25,27]. PSM ≥ 3 mm in our cohort showed to be an independent predictor for BCR and, these patients were at higher risk for recurrence and had lower BFS rates than patients with NSM or PSM < 3 mm. Multifocality of the PSM has also been proposed as an adverse feature in PSM [16,25,41]. Our results agree with this statement and multifocality conferred a higher risk for BCR. In addition, the presence of higher Gleason at the margin (Gleason > 3) seems to be a strong feature of aggressiveness at the margin and it is proposed as an independent factor of poor prognosis in a recent meta-analysis [36]. However, in our study, we couldn’t reproduce the associated higher risk for BCR of margins with Gleason > 3 in comparison with NSM or PSM with Gleason = 3. Probably, due to the low representation of this feature in our series. 

After assessing the impact of adverse characteristics individually on the risk of BCR, we wanted to investigate the combined effect of these unfavourable features to identify those patients with PSM that are at higher risk for BCR. Previous literature about PSM has not stated the simultaneous effect of adverse length, focality and Gleason at the margin. In our study, PSM ≥ 3 mm and multifocal strongly predicted BCR, associated higher risk of BCR compared to favourable margins and showed significant lower BFS rates in the survival analyses. The coexistence of all three adverse features in the PSM (≥3 mm, multifocal and Gleason at margin > 3) is a strong predictor for BCR (HR 5.98; 95% CI 1.86–19.24, *p* = 0.003). However, we could not prove that margins featuring all three adverse features are associated with increased BCR risk compared to favourable margins (HR 2.99; 95% CI 0.99–9.02, *p* = 0.052). This late finding should be interpreted considering that the number of patients with PSM featuring all adverse characteristics in our cohort was quite small (*n* = 6). Nevertheless, the survival analyses conferred significant lower BFS to this type of margin compared to favourable ones. The previous findings strongly support the statements of the International Society of Urological Pathology (ISUP) Consensus Conference that advocates to routinely report PSM length, location, focality and Gleason in the pathology report after radical prostatectomy [42].

On the other hand, including adverse PSM features beyond margin status (positive vs negative) in our multivariable prediction model for BCR did not meaningfully enhance the model’s discrimination.

The commonest locations for PSM were the posterior (31.5%) and the apical region (25.8%), in agreement with other series [2,8,23]. Posterior margins and their close relation with neurovascular tissue involve a balance between preserving as much neurovascular bundle as possible and the risk of leaving tumour and endangering oncologic control. Furthermore, positive posterior margins, apparently, are associated with a higher risk for BCR in comparison with other locations [43]. Thus, neurovascular bundle preservation and the plane of dissection, between intra- vs. inter- vs. extrafascial approach, should be performed according to tumour characteristics. On the other hand, the apical region represents a challenging dissection location due to its highly variable anatomy and its relationship with structures such as the dorsal venous complex, erectile nerves and the urethral sphincter. However, apical PSM are suggested to be, on some occasions, false-positive surgical margins due to the absence of capsule at the prostatic apex, causing errors when analysing the specimens [2]. The impact of apical margins on oncologic outcomes remains uncertain. Both posterior and apical PSM showed to be significant predictors of BCR in our study, while other locations did not, in some measure, due to low representation of other PSM locations in our population. Apical PSM were associated with higher HRs in our cohort in comparison with posterior margins and PSM overall in contrast with other series [44]. To minimise the risk of PSM during radical prostatectomy and, therefore, the risk of BCR, an intraoperative frozen section (IFS) has been proposed. However, its use is still controversial [45].

Lastly, we tested the effect of adverse PSM in patients with favourable pathology findings (pT2 and ISUP < 4). Trials assessing the effect of adjuvant radiation therapy (ART) after RP included mostly patients with locally advanced disease [12,13,14]. ART in patients with PSM, despite favourable pathology findings as organ-confined disease and favourable ISUP, in great measure, would lead to overtreatment and toxicity. Therefore, it would be interesting to identify a subgroup of patients with adverse PSM that, regardless of favourable findings in the prostatectomy specimen, could benefit from ART or closer surveillance and early salvage radiotherapy and differentiate from those whose PSM will not alter the history of the disease. However, evidence is still lacking. Chapin et al. found a subset of patients with PSM > 1 mm or with Gleason at the margin > 3 with organ-confined disease that associated higher risk for BCR and could benefit from closer surveillance [26]. Similarly, Preisser et al. stated that PSM ≥ 3 mm or with Gleason > 3 in pT2 specimens associated with worse BFS and should be monitored closely for early salvage radiotherapy [27]. In our study, patients with favourable pathology after RARP (pT2 and ISUP < 4) and PSM were at higher risk for BCR (Table 4). Adverse length, focality and Gleason at the margin were also predictors for BCR when analysed separately. Patients featuring adverse length and focality at the PSM associate even higher HRs for BCR. In our series, no patients with favourable pathology after RARP had the coexistence of the three adverse features in their margins.

Taken together, from a clinical standpoint and with the current evidence is still early to establish a proper PSM risk stratification and determine further management of these patients after RP. Previous randomised trials showed improved BCR-free survival with immediate adjuvant radiation therapy in patients with adverse pathology findings, including PSM [12,13,14]. However, its impact on clinical recurrence or stronger oncologic endpoints is controversial. A percentage of patients with adverse pathology and PSM who receive ART would never have experienced disease relapse, and this leads to toxicity and overtreatment. Moreover, up to now, merely PSM do not justify additional treatment [11,16]. Hence, three trials are assessing the role of early salvage radiotherapy in comparison with ART in patients with locally advanced disease and with PSM [18,19,20]. A meta-analysis that included these trials showed that ART does not improve event-free survival in comparison with early salvage radiotherapy [21]. Therefore, close surveillance and early salvage radiotherapy as a standard of care for this group of patients would contribute to avoiding immediate toxicity after RP and applying additive therapies only to those that really develop BCR without compromising oncologic outcomes. These findings question previous literature that suggested ART as an option for patients with PSM. Besides, trials assessing the role of ART or early salvage radiotherapy consider all PSM as an equal; however, this study and current evidence support the hypothesis that not all PSM harbour the same risk for recurrence. Finally, from our standpoint, and being cautious due to lack of evidence, patients with PSM featuring adverse characteristics, and considering that this involves a higher risk for BCR, could benefit from closer surveillance. Nonetheless, further studies should assess if adverse PSM have an impact on clinical recurrence that can justify additional treatment. 

We acknowledge some limitations in the present study. First the retrospective nature of the study, as almost all studies regarding this topic. Second, the median follow-up of the cohort was relatively short to assess stronger oncologic outcomes besides BCR. Third, the low representation of some adverse features in the PSM, especially in the case of Gleason at the PSM, that was not reported in the pathology report until 2017. Fourth, the limited number of patients in our cohort with PSM harbouring the combination of all adverse features regarding length, focality and Gleason at the margin. Due to this, a cautious interpretation of the results obtained in that case should be taken. Fifth, even though prostatectomy specimens were analysed by uropathologists and surgical margins definition is based on a consensus, thermal energy, electrocautery or manipulation during surgery could artefact the margins report.

## 5. Conclusions

PSM are an independent predictor for BCR, however, not all PSM carry the same risk for recurrence. PSM with adverse characteristics regarding length and focality are associated with a greater risk of BCR and worse BCR-free survival compared to favourable surgical margins. Nevertheless, subclassifying PSM with adverse features did not enhance the model’s predictive performance in our cohort. Future studies assessing if patients with unfavourable PSM are at higher risk for clinical recurrence and would benefit from closer surveillance and additional treatment are needed.

## Figures and Tables

**Table 1:** Characteristics of patients that underwent RARP between 2015–2020 were stratified by margin status.

Variable	All Patients *n* = 333	Negative Surgical Margins *n* (%) = 209 (62.8)	Positive Surgical Margins *n* (%) = 124 (37.2)	*p* Value
Age (years), median (IQR)	65 (59–68.5)	65 (60–69)	65 (59–68)	0.328
BMI (kg/m^2^), median (IQR)	27 (25–29.75)	26 (25–29)	27.50 (25–30)	0.021
Preop PSA (ng/mL), median (IQR)	6 (4.8–8.5)	5.98 (4.74–8.0)	6.17 (5.02–9.22)	0.088
Surgery time (min), median (IQR)	170 (140–210)	170 (140–210)	175 (140–205)	0.611
Prostate volume (gr), median (IQR)	50 (39–65)	55 (40–65)	42.5 (34.25–60.00)	<0.001
pT, n (%)				0.020
pT2	250 (75.1)	167 (79.9)	83 (67.0)	
pT3a	70 (21)	34 (16.3)	36 (29.0)	
pT3b-T4	13 (3.9)	8 (3.8)	5 (4.0)	
pN, n (%)				0.735
Nx	244 (73.3)	156 (74.6)	88 (71.0)	
N0	87 (26.1)	52 (24.9)	35 (28.2)	
N1	2 (0.6)	1 (0.5)	1 (0.8)	
Pathological ISUP, n (%)				0.149
1	76 (22.8)	54 (25.8)	22 (17.7)	
2–3	223 (67.0)	137 (65.6)	86 (69.4)	
4–5	34 (10.2)	18 (8.6)	16 (12.9)	
Positive surgical margins, n (%)	124 (37.2)			
ECE, n (%)	83 (24.9)	42 (20.1)	41 (33.1)	0.008
SVI, n (%)	13 (3.9)	8 (3.8)	5 (4.0)	0.926
Follow-up (months), median (IQR)	34.5 (26.3–47.0)	33.7 (25.0–46.6)	36.62 (28.06–48.04)	0.275
BCR, n (%)	60 (18.0)	20 (9.6)	40 (32.3)	<0.001

RARP = Robotic-assisted radical prostatectomy; IQR = Interquartile range; BMI = Body mass index; PSA = Prostate-specific antigen; ISUP = International Society of Urological Pathology; PSM = Positive surgical margins; ECE = Extracapsular extension; SVI = Seminal vesicle invasion; BCR = Biochemical recurrence.

**Table 2:** Characteristics of the positive surgical margins.

Variable	Positive Surgical Margins
Length of PSM (mm), median (IQR)	3.5 (2–6)
Length of PSM (mm), n (%)	
<3 mm	49 (39.5)
≥3 mm	75 (60.5)
Focality, n (%)	
Unifocal	91 (73.4)
Multifocal	33 (26.6)
Location, n (%)	
Apex	32 (25.8)
Posterior	39 (31.5)
Lateral	8 (6.5)
Anterior	8 (6.5)
Vesical	4 (3.2)
Multiple	33 (26.6)
Gleason Grade at PSM, n (%)	
3	74 (59.7)
4	20 (16.1)
5	2 (1.6)
Unknown	28 (22.6)

PSM = Positive surgical margins; IQR = Interquartile range.

**Table 3:** Univariable and multivariable* Cox regression models predicting biochemical recurrence after RARP and the role of PSM and their adverse characteristics. Multivariable models’ discrimination with optimism-corrected c-indices are shown.

Variable	Univariable Analysis		Multivariable Analysis*		
	HR (95% CI)	*p*-Value	HR (95% CI)	*p*-Value	c-Index
PSM (vs. neg)	3.74 (2.18–6.41)	<0.001	3.71 (2.13–6.47)	<0.001	0.738 (0.66–0.80)
PSM ≥ 3 mm (vs. neg)	4.66 (2.63–8.27)	<0.001	4.51 (2.49–8.17)	<0.001	0.751 (0.69–0.82)
(vs. favourable margins)	3.68 (2.21–6.13)	<0.001	3.50 (2.05–5.95)	<0.001	
PSM multifocal (vs. neg)	4.23 (2.01–8.92)	<0.001	4.16 (1.90–9.09)	<0.001	0.740 (0.68–0.81)
(vs. favourable margins)	2.40 (1.24–4.66)	0.009	2.18 (1.09–4.37)	0.028	
PSM with GG > 3 (vs. neg)	6.06 (2.83–12.99)	<0.001	3.02(1.33–6.86)	0.008	0.739 (0.66–0.82)
(vs. favourable margins)	3.98 (1.98–7.98)	<0.001	2.00 (0.94–4.27)	0.072	
PSM ≥ 3 mm and multifocal (vs. neg)	4.75 (2.20–10.26)	<0.001	4.66 (2.08–10.42)	<0.001	0.740 (0.68–0.81)
(vs. favourable margins)	2.69 (1.35–5.34)	0.005	2.43 (1.18–4.99)	0.016	
PSM ≥ 3 mm, multifocal and GG> 3 (vs. neg)	15.17 (4.42–39.27)	<0.001	5.98 (1.86–19.24)	0.003	0.740 (0.68–0.80)
(vs. favourable margins)	6.99 (2.49–19.61)	<0.001	2.99 (0.99–9.02)	0.052	

* Adjusted for preoperative PSA, pathologic stage (pT) and pathologic ISUP. RARP = Robotic-assisted radical prostatectomy; HR = Hazard ratio; CI = Confidence Interval; PSM = Positive surgical margins; neg = negative surgical margins; GG= Gleason Grade at the margin; favourable margins = PSM without adverse characteristics and NSM.

**Table 4:** Cox regression model predicting biochemical recurrence after RARP and the role of PSM characteristics in patients with favourable pathology after RARP (pT2 and ISUP < 4).

Variable	HR (95% CI)	*p* Value
PSM (vs. neg)	5.05 (2.55–11.90)	<0.001
PSM ≥ 3 mm (vs. neg)	6.82 (2.95–15.79)	<0.001
PSM multifocal (vs. neg)	7.06 (2.34–21.29)	0.001
PSM with Gleason at margin > 3 (vs. neg)	8.20 (2.50–26.92)	0.001
PSM ≥3 mm and multifocal (vs. neg)	7.12 (2.17–23.33)	0.001

RARP = Robotic-assisted radical prostatectomy; HR = Hazard ratio; CI = Confidence Interval; PSM = Positive surgical margins; neg = negative surgical margins.

## Data Availability

Not applicable.

## Supplementary Materials

The following are available online at https://www.mdpi.com/article/10.3390/biomedicines10081911/s1, Table S1: Univariable Cox regression models predicting biochemical recurrence after RARP, Table S2: Univariable and multivariable Cox regression models predicting biochemical recurrence after RARP and the role of PSM location.
