# Supplementary material for: The Effect of Adverse Surgical Margins on the Risk of Biochemical Recurrence after Robotic-Assisted Radical Prostatectomy
Source: Biomedicines. 2022 Aug 7;10(8):1911. doi: 10.3390/biomedicines10081911 (PMC9405399; doi:10.3390/biomedicines10081911)
Supplement: Supplementary file 1 [file biomedicines-10-01911-s001.zip › Supplementary files.pdf]

Supplementary files

Table S1. Univariable Cox regression models predicting biochemical recurrence after RARP

Variable	Univariable Analysis	
	**HR (95% CI)**	***p*-value**
Preoperative PSA ≥10 (vs <10)	1.35 (0.72-2.54)	0.353
ISUP 2-3(vs 1)	3.77 (1.35-10.54)	0.011
ISUP ≥4 (vs 1)	12.81 (4.24-38.72)	<0.001
pT3-T4 (vs T2)	1.87 (1.10-3.19)	0.021
SVI (vs no SVI)	2.87 (1.23-6.68)	0.014
PSM (vs neg)	3.74 (2.18-6.41)	<0.001
PSM <3mm (vs neg)	2.46 (1.18-5.15)	0.017
PSM ≥3mm (vs neg)	4.66 (2.63-8.27)	<0.001
PSM unifocal (vs neg)	3.59 (2.03-6.35)	<0.001
PSM multifocal (vs neg)	4.23 (2.01-8.92)	<0.001
PSM with Gleason at margin 3 (vs neg)	3.15 (1.69-5.88)	<0.001
PSM with Gleason at margin>3(vs neg)	6.06 (2.83-12.99)	<0.001
PSM<3mm or unifocal	3.50 (1.99-6.17)	<0.001
PSM≥3mm and multifocal (vs neg)	4.75 (2.20-10.26)	<0.001
PSM<3mm or unifocal or Gleason at margin 3	3.22 (1.87-5.56)	<0.001
PSM≥3mm, multifocal and Gleason at margin>3 (vs neg)	15.17 (4.42-39.27)	<0.001

RARP = Robotic-assisted radical prostatectomy; HR = Hazard ratio; CI = Confidence interval; PSA = Prostate-specific antigen; SVI = Seminal vesicle invasion; PSM = Positive surgical margins; neg = negative surgical margins.

Table S2. Univariable and multivariable* Cox regression models predicting biochemical recurrence after RARP and the role of PSM location

Variable	Univariable Analysis		Multivariable Analysis*	
	**HR (95% CI)**	***p*-value**	**HR (95% CI)**	***p*-value**
Apex (vs neg)	4.38 (2.17-8.83)	<0.001	4.88 (2.37-10.03)	<0.001
Posterior (vs neg)	3.41 (1.66-7.00)	0.001	3.42 (1.64-7.12)	0.001
Lateral (vs neg)	1.31 (0.18-9.73)	0.795	1.36 (0.18-10.28)	0.765
Anterior (vs neg)	3.64 (0.85-15.64)	0.083	2.42 (0.55-10.66)	0.243
Vesical (vs neg)	3.48 (0.47-26.00)	0.224	3.64 (0.46-28.58)	0.219
Multiple (vs neg)	4.23 (2.01-8.92)	<0.001	4.16 (1.90-9.09)	<0.001

*Adjusted for preoperative PSA, pathologic stage (pT) and pathologic ISUP.

RARP = Robotic-assisted radical prostatectomy; HR = Hazard ratio; CI = Confidence Interval; PSM = Positive surgical margins; neg = negative surgical margins.
